# Structure of respiratory complex I – An emerging blueprint for the mechanism

**DOI:** 10.1016/j.sbi.2022.102350

**Published:** 2022-03-19

**Authors:** Domen Kampjut, Leonid A. Sazanov

**Affiliations:** a MRC Laboratory of Molecular Biology, Cambridge CB2 0QH, UK; b Institute of Science and Technology Austria, Am Campus 1, 3400 Klosterneuburg, Austria

## Abstract

Complex I is one of the major respiratory complexes, conserved from bacteria to mammals. It oxidises NADH, reduces quinone and pumps protons across the membrane, thus playing a central role in the oxidative energy metabolism. In this review we discuss our current state of understanding the structure of complex I from various species of mammals, plants, fungi, and bacteria, as well as of several complex I-related proteins. By comparing the structural evidence from these systems in different redox states and data from mutagenesis and molecular simulations, we formulate the mechanisms of electron transfer and proton pumping and explain how they are conformationally and electrostatically coupled. Finally, we discuss the structural basis of the deactivation phenomenon in mammalian complex I.

## Introduction

Complex I catalyzes NADH oxidation and quinone reduction to quinol, couples this reaction to pumping of four protons across the membrane, and is thus a major contributor to the proton motive force (pmf) driving ATP synthesis. Complex I is fully reversible and can also catalyse the reverse electron transport (RET), when pmf and quinol drive NAD^+^ reduction [1]. Complex I was the last one of the respiratory enzymes to be characterised structurally [2–4] but the first structures did not immediately suggest a clear mechanism by which proton pumping and redox machinery are coupled over a distance of over 200 Å. In recent years, however, structures of complex I, often in different substrate- or inhibitor-bound states, have become available from all of the traditional model systems, including several mammalian species, bacteria (*Thermus thermophilus*, *Escherichia coli*), fungi (*Yarrowia lipolytica*) and plants, giving new insights into the mechanism [2,5–9].

**Figure 1:**
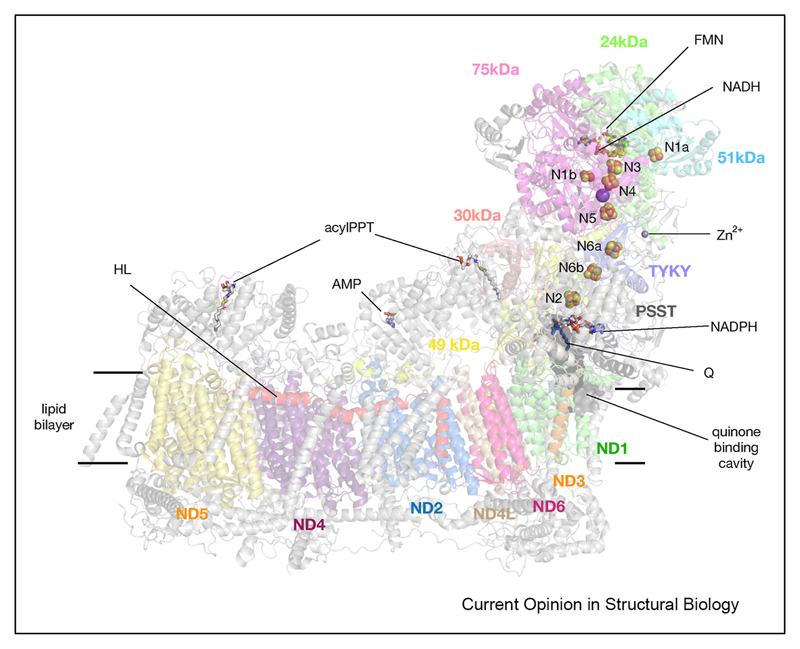
Structure of mammalian complex I. Structure of the mammalian complex I with 14 core subunits coloured and annotated and the remaining supernumerary subunits shown in grey. Substrates and cofactors are annotated.

The general structure of complex I is conserved across species and consists of fourteen core subunits equally divided between the peripheral arm (PA) responsible for electron transfer and the membrane arm (MA) responsible for proton pumping (Figure 1). In eukaryotes and some bacteria, additional supernumerary subunits exist which are believed not to be involved in the catalytic reaction but are necessary for the correct assembly and functioning of the complex I [10–13]. Mammalian complex I thus consists of 45 polypeptides with a total mass of ~ 1 MDa and along with respiratory chain complexes III and IV forms even larger “supercomplexes” [14,15]. Evolutionarily, the membrane domain is related to the Mrp Na^+^/H^+^ antiporter [16,17], while the peripheral arm is related to the NiFe hydrogenase [18]. Structures of several evolutionarily closely related enzymes to complex I, such as Ndh, Mbh/Mbs and Mrp complexes, which share many homologous subunits, have also been solved recently [16,19–22] and provide further evidence for the generalisable mechanism.

## Peripheral arm of complex I and electron transfer reaction

In the peripheral arm of the enzyme, electrons from NADH are accepted by FMN (as a hydride) and then passed along a chain of iron-sulfur clusters by electron tunnelling to the final acceptor quinone. There are eight or nine FeS clusters, depending on the species, but only seven of them lie on the main pathway connecting the NADH and quinone. N1a lies off-path “upstream” of FMN, but close enough to be able to accept electrons, so its role could be to temporarily store an electron to prevent flavosemiquinone formation and reduce ROS generation [23]. The ninth FeS cluster N7 exists only in bacteria and is located too far off the main path (~ 20 Å) to be reducible and is probably an evolutionary vestige [2,5]. Two clusters are binuclear (N1a and N1b) while the rest are tetranuclear. The seven conserved core subunits of the PA can be assigned to the N- (NADH-binding) and Q- (quinone-binding) modules (Table 1) [24]. In mammals, 31 supernumerary subunits form a shell around the core and have putative structural and regulatory roles which have recently been reviewed [13].

Due to electrostatic interactions [25], in the steady state situation with an excess of NADH, not all of the FeS clusters are reduced but instead one electron resides approximately on every other FeS cluster [26]. Most of the reducible clusters are roughly equipotential with the NAD^+^/NADH pair at around —250 mV, and the only cluster with a higher potential is the final N2 cluster at around — 150mV [25,27,28]. Since the largest drop in redox potential occurs between N2 and the quinone/quinol pair (+100 mV), the crucial energy-releasing step in the reaction is therefore quinone reduction or protonation or perhaps even its release out of the binding cavity as the potential of the Q/QH_2_ pair bound near N2 is likely similar to the N2 potential [28]. This has been confirmed by real-time measurements of electron transfer reaction in the peripheral arm, which furthermore ruled out the involvement of a long-lived semiquinone radical in the mechanism [29].

The reduction of the isolated PA [30] by NADH was found to elicit intriguing changes in the coordination of cluster N2, which suggests that the unique coordination of this cluster by two consecutive cysteines affords some flexibility in this area. However, such changes were not observed in the intact enzyme [31], suggesting that N2 coordination is to some extent stabilised in the presence of MA, although the remaining flexibility possibly helps to enable conformational changes observed during turnover. Reduction of N2 is fast and not rate-limiting [29] and so is unlikely to drive the proton pumping machinery by itself. Consistently, NADH or NAD^+^ in the absence of quinone or quinone-site inhibitors are not able to induce large conformational changes in *T*. *thermophilus* enzyme [31]. Nevertheless, prolonged incubation of the *T*. *thermophilus* and ovine complex I with NADH induced changes in the 49 kDa and ND1 loops, respectively, which could help eject quinol out of the cavity [31,32].

## Membrane arm and the mechanism of proton pumping

Membrane arm (MA) of complex I contains seven core subunits which are central to the catalytic reaction (Table 1) [33]. ND1 subunit (bovine nomenclature) is the closest to the PA and forms part of the PA—MA interface. It is followed by three small subunits, ND3, ND6 and ND4L, which form part of the E-channel, containing key glutamates [2]. Finally, the complex contains three homologous antiporter-like subunits (ALS), ND2, ND4 and ND5, which were historically postulated to pump one proton each [2,33]. A central axis of charged residues connects these subunits, suggesting a way of long-distance communication along the MA [2,34,35].

The E-channel and the ND1 subunit are now understood to play a more important role in the coupling of the reaction rather than in proton pumping itself, which happens in the ALS. ALS share a cation/proton antiporter (Mrp) fold of two inverted symmetric 5 TMH repeats forming one half of the putative proton translocating channel each. Both halves contain a conserved lysine residue, LysTM7/12 (or GluTM12 in case of ND4), connected by a central LysTM8 (or histidine in the case of ND5), that are key to proton pumping. In each ALS, LysTM7 forms an ion pair with a conserved TM5 glutamate, which is thought to modulate the pK_a_ of the lysine [33]. All of these key residues sit on breaks in TM helices, which likely renders the central hydrophilic axis flexible. This has led to various mechanistic proposals involving conformational changes [35,36], perhaps aided by the long traverse helix HL from ND5 (Figure 1) [2,33]. An electrostatic wave mechanism proposed in 2018 based on MD simulations involves coordinated forward and backward waves of conformational changes and charge exchange from quinone site to the tip of ND5 subunit [35]. An earlier version of a similar model was based on mutagenesis studies of conserved residues of the central axis [37,38]. While the critical role of lysine residues proposed has stood the test of time, the notion that the doubly charged quinone directly electrostatically interacts with lysines in antiporters, causing the forward and backward electrostatic waves appears to be incompatible with the recent work.

**Figure 2:**
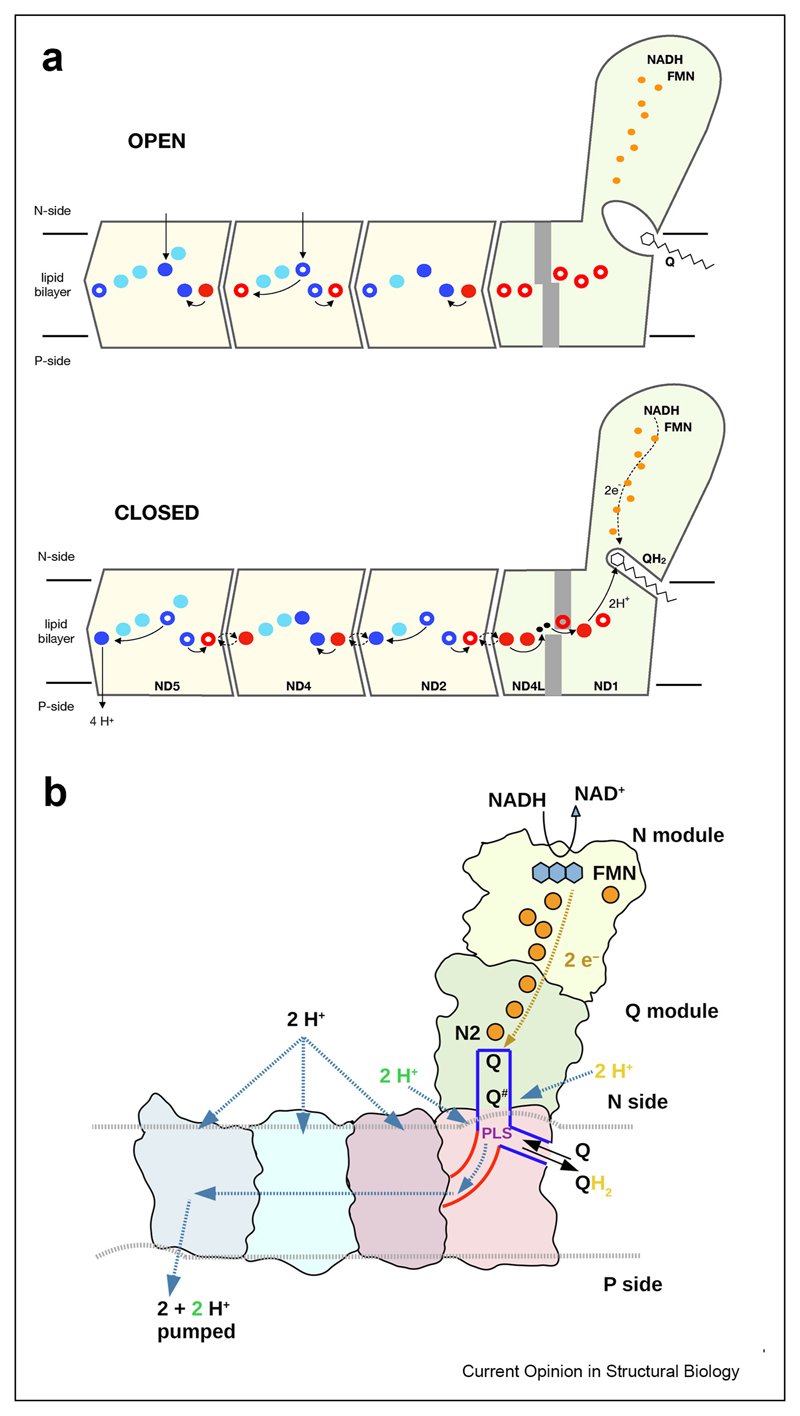
Mechanism proposal for complex I. **a**. A schematic representation of complex I divided into the major functional domains illustrates the recently proposed mechanism with further details available in Ref. [30]. In the open state, the Q cavity is sufficiently enlarged to allow free passage of quinone in and quinol out of the cavity. The water wire between the PA and MA is broken by the rotation of ND6_TMH3 (in grey). In the closed state, quinone can bind in the deep site and accept electrons, leading to the relocation of chemical protons from the E-channel/ND2 interface via the newly established water wire connection into the tightly enclosed Q cavity. This creates a strong negative charge near ND2 GluTM5/LysTM7 pair, initiating a series of protonation/de-protonation events (indicated by arrows), driven by electrostatic interactions. Eventually this leads to the expulsion of four protons into IMS (P-side) via ND5. The coloured circles represent glutamate or aspartate residues (red), lysine residues (blue) or histidine residues (cyan) in a same relative spatial arrangement as in the mammalian complex I structure. Full circles denote charged residues (negatively in the case of Glu/Asp and positively in the case of Lys) and empty circles denote neutral residues. Charge of histidines is not clear from current structures. **b**. An alternative mechanistic proposal from Parey et al. [6] (reproduced with permission). Note the presence of a long-lived negatively charged intermediate Q# and different proton paths for the chemical and pumped protons. In short, it is proposed that after transfer of each electron to Q, Q# moves to the second site near a putative proton-loading site (PLS) and accepts a chemical proton (yellow) from the matrix (cycle repeats twice). Each protonation of Q^#^ leads to injection of pumped proton (green) from the PLS into E-channel (red) and further towards antiporters. For each Q to QH2 reaction, two pumped protons are injected via PLS, driving two more pumped protons via antiporters. See Ref. [6] for details.

The absence of any conformational changes in the ALS in recent structures, including during turnover, rule out the conformationally driven mechanisms and instead strongly support a purely electrostatic mechanism for the ALS [32]. Another surprise of the recent structures is the unique hydration pattern of the ALS, with the ND5 being much more hydrated at the intermembrane space (IMS) side (or P-side) than the ND2/ND4, first observed in mammalian enzyme [32] and later in yeast [6]. This unique hydration profile is consistent also with both available Ndh structures [21,22] and with molecular dynamics simulations [39,40], although conclusions differed in one case [40]. This suggests that ND2 and ND4 do not possess viable hydrated proton pathways leading towards the P-side of the membrane and only ND5 has a full proton input and output pathway. ND4 appears also to have proton input pathway from N-side, while ND2 probably not, perhaps depending on the species. The E-channel is dry on both sides of the membrane, ruling out its involvement in proton pumping. This suggests an exciting and unexpected possibility that all the protons are ejected through the ND5, with three to four histidines in ND5 possibly serving as temporary proton storage [6,32] (Figure 2). Whether the protons are ejected simultaneously or sequentially and where exactly they are taken up from remains to be determined. ND5-only proton pumping model is consistent with very distinct sequence conservation pattern of this subunit (Supplementary text in Ref. [32]) and elegantly explains many known mutants of ND5 which completely abolish proton pumping [41,42].

## Mechanism of quinone reduction and coupling

The most intricate part of the complex I and the last one to be resolved structurally is the PA—MA connection [2]. It contains a quinone binding site which is a ~25 Å long channel formed mainly by the flexible loops of the 49 kDa, ND1 and PSST subunits. Further two flexible loops of ND3 and ND6 subunits form the rest of the PA/ MA interface. Conformations of these five loops control the shape and accessibility of the quinone cavity and the overall angle between the PA and MA. Broadly speaking, complex I can exist in the closed state, in which the angle between the PA and the MA is smaller and the quinone cavity is long, narrow and tightly isolated from the bulk solvent. In the open state the angle between the two arms increases and the quinone binding cavity widens and becomes accessible to the bulk solvent [6,32,43].

The quinone head can bind at two distinct sites in the quinine binding cavity, the shallow, Q_s_, and the deep, Qd, site. The shallow site was first predicted by MD [44,45] and later confirmed structurally [32,46]. Quinone redox chemistry can only happen at the Q_d_ site as the Q_s_ is too far away from the N2 cluster. Electrons get transferred to quinone at the Qd site in quick succession such that no long-lived quinone or semiquinone radical intermediate exists [29,47] and any mechanism proposals featuring such intermediates thus appear unlikely [5,6]. Electron transfer is then quickly followed by two protons which initially come from the 49 kDa triad His59/Asp160/Tyr108 as shown structurally [32].

In the open state, any quinones, either native or supplemented, have been observed to bind near the Q_s_ site only, while binding near the Q_d_ site was only observed in the closed states [32]. This suggests that access to the Q_d_ site is controlled by the global conformation of complex I and can only happen in the closed state, when the cavity is tightly sealed from the bulk solvent. This ensures that chemical protons for the quinone reaction come from the E-channel glutamates via a water wire, creating double negative charge at the E-channel/ND2 interface, which initiates a series of electrostatic interactions within ALS and proton pumping [32]. Opening of the cavity in the open state, on the other hand, helps push the reduced quinol out of the cavity and allows quinone exchange as the entrance in the closed state appears too narrow to allow passage of the quinone head group [48].

Both quinone binding sites can also bind inhibitors piericidin A and rotenone [32,49], although binding of rotenone at Q_s_ appears to be possible only in the open conformation. Most other inhibitors, piericidin A, aureothin, pyridaben, favour the Q_d_ site in T *thermophilus* as well [31], although a new inhibitor which favours the Q_s_ site has been found recently [50]. This supports the idea that binding of quinone at the Q_d_ site favours the closed state while diffusion of quinol out of the cavity favours the open state and disordering of the quinone site loops.

Comparisons of high resolution structures of open and closed states of complex revealed large scale reorganisations within ND1 and leading all the way to the first ALS, ND2. The most striking of these is the rotation of the TM3 of ND6 which acts as a gate for the water wire connection between antiporters and the quinone binding site [32]. In the closed state there is no π-bulge in TMH3 and the water wire is established, while in the open state, the formation of π-bulge disrupts the water wire and effectively isolates the quinone binding site from the antiporters by bulky amino acids. This prevents the establishment of a futile cycle and ensures that quinone is not reduced without concomitant proton pumping and that no proton leak occurs. The resulting mechanism is thus an unexpected combination of large conformational changes around Q cavity/E-channel and electrostatic interactions within ALS (Figure 2a).

This proposal has been questioned by the fact that TM3 rotation has so far only been observed in the closed mammalian state. However, as discussed in detail in the next section, the closed state as a high-energy intermediate is easier to observe in some species than in others, especially those that have a low deactivation barrier (*Y*. *lipolytica*). In *Y*. *lipolytica* under turnover conditions only one conformational state (instead of at least two expected during turnover) was observed, resembling the open state of ovine enzyme as similar areas (such as ND3 loop) are disordered [6]. On the other hand, as-purified mouse enzyme, with high deactivation barrier, shows mostly closed state [51]. Since minor 3D classes (such as 10% closed in Ref. [32]) are challenging to classify out with standard techniques, it is possible that with our focus-reverse-classify approach, developed specifically for this purpose [14,32], minor classes may be revealed also in these datasets (closed state in *Y. lipolytica* or open in mouse). In *T. thermophilus* quinone also induced conformational changes that extended along ND1 into the E channel, along with shifts in ND6 TMH3, but barring a full rotation, likely because turnover conditions have not yet been imaged in cryo-EM in these species [31]. A recent mutagenesis experiment indirectly supports the proposal [32]. A linkage of ND3/Cys40 and PSST/ Gln133Cys (separated by 11 Å) introduced to study the role of the ND3 loop motion in the mechanism, inadvertently, most likely displaced ND3 loop and opened a hole into the Q cavity in the closed conformation [52]. Thus, the observed decoupled proton translocation from redox reaction is consistent with the mechanism [32], because Q could now be protonated by bulk water instead of protons from the E-channel.

An alternative mechanism proposed recently on the basis of high-resolution structure of *Y. lipolytica* enzyme, in common with our proposal, suggests that all protons are pumped out via ND5 (Figure 2b). However, the rest of the mechanism appears less likely as it involves long-lived semiquinone radicals which have not been observed experimentally and also it does not provide a plausible mechanism for gating at the Q site since chemical protons for quinone reduction can come freely into Q cavity from the matrix [5,6]. Therefore the possibility for the direct functional link with the central MA axis is lost (Figure 2b).

Thus, the recent structural and mechanistic models finally explain a number of enigmatic features of complex I mechanism and falsify some of the earlier more theoretical proposals for coupling. Mechanisms which involve simultaneous binding of two quinones are not likely to work simply because the quinone cavity is too narrow to house more than one molecule of quinone at a time [28,53]. Electrostatic wave mechanisms on the other hand do not predict the important role of quinone cavity rearrangement and ND6 TM3 in the coupling mechanism [35,37]. Recently MD was used to suggest that ND6 TM3 rotation breaks up the water wire in the deactive state of the complex [54], a feature which was demonstrated experimentally much earlier both for deactive and open states [32]. Another recent theoretical paper described a structurally vague mechanism with a charged “piston” which may resemble the ND6 TM3 action [55]. The rate equations were solved numerically, suggesting that the mechanism is plausible with a reasonable set of parameters.

## Mechanism of deactivation

Eukaryotic complex I can exist in a deactive conformation which is a catalytically inactive state of the enzyme originally defined by the NEM-sensitivity of the exposed Cys39 of ND3 loop [56,57]. Deactivation could be important physiologically to prevent RET and ROS formation under ischaemia as mammalian complex I slowly becomes deactivated at elevated temperatures (30-37 °C) in the absence of substrates and reverts slowly (1-4 min ^−1^) back to the active state in the presence of substrates [58]. In *Y. lipolytica*, both the deactivation and reactivation proceed much faster and do not require elevated temperatures [59]. Bacterial enzyme does not show a pronounced deactive state, however, *E*. *coli* enzyme can enter a so-called “resting” inactive state, from which it can recover within 1—2 s of turnover [60].

It has been argued that the open conformation of complex I corresponds to the deactive state in mouse and bovine complex because the proportion of the apparently “open” complex I in the sample increased upon incubation at 37 °C without substrates [51,61]. However, this simplistic explanation is in conflict with the fact that open and closed conformations of complex I were observed in active preparations of ovine complex I [14,32,62] and does not explain why a mixture of open and closed complex were also observed in the deactive mouse and bovine preparations that led to the notion of the open complex I being deactive [51,61]. Furthermore, cryo-EM experiments under turnover conditions, essential to observe full conformational space of complex I, were not yet performed for bovine or mouse enzyme. MD simulations suggest that unfolding and refolding of mobile loops which control the opening and closing can happen on a millisecond timescale and should not limit the reaction [45]. Crucially, biochemically prepared fully deactive ovine complex I exhibits extensive specific rearrangements, including relocation of the entire ND6 TM4, conclusively showing that the deactive state is a specific extreme conformation of the open state and is not equivalent to it [32]. Since the active open state also has a disordered ND3 loop and thus an exposed Cys39, NEM sensitivity assay estimates in fact a proportion of the combined open and deactive states, rather than just the deactive state. The only reliable biochemical assay for deactive state is thus the delay in the development of activity upon addition of substrates [32].

The most recent work on the ND6_P25L mutant of mouse complex I also confirms that the open state is a catalytic intermediate, although this has not been interpreted by the authors as such [63]. The mutant behaves very similarly to ovine complex I: even when pre-activated with NADH, it still remains ~60% susceptible to NEM. We suggest that the mouse mutant is simply a stabilised open state intermediate, and not, as the authors suggest, an “active complex with deactivelike characteristics”. Finally, recent biochemical work on bovine complex I also supports the notion that Cys39 of ND3 loop is exposed during respiration [64].

Complex I thus exists in an equilibrium between closed and open states, with the exact position of the equilibrium depending heavily on the environmental parameters and the species in question. Discovery of the profoundly open state in *E*. *coli* complex I (which probably represents the “resting” state) further underlines this view as the *E*. *coli* complex I is catalytically competent without large delay in activity [5].

## Conclusions and future directions

To conclude, significant advances have been made in understanding of the complex I in the recent years. We now understand the detailed mechanism of quinone reduction and its complex binding pattern within the flexible cavity. We also have increasingly strong evidence for the significance of the closed and open conformations in the catalytic cycle, not only in the deactivation of the complex. Finally, recent structures and MD simulations have challenged the long-held view that there are four proton pumps in the complex, each responsible for translocation of one proton.

Structures of complex I from different species and from a wider family of complex I-related enzymes are consistent with these findings and show a remarkable versatility of the conserved complex I modules in adaptation to different substrates and stoichiometries of proton pumping. Core principles of proton pumping and coupling however, seem to remain surprisingly conserved.

The principles of mechanism outlined here are undoubtedly going to be further refined in future studies involving structural approaches, biophysics, mutagenesis and MD. Novel avenues for studying complex I structurally using time-resolved cryo-EM approaches or in liposomes under PMF conditions are also going to refine our knowledge on the complex I mechanism.

## Note added in Proof

A recent publication [67] has claimed to overturn our mechanism of complex I [32] and proposed an alternative coupling mechanism, based on a permanently bound ubiquinone shuttling electrons from the deep to the shallow binding site, where they get transferred to a hypothetical loosely bound external molecule of ubiquinone. This proposal is inconsistent with current knowledge on complex I and the authors do not present any experimental evidence for the binding of an external quinone. On the contrary, there are no visible cavities and no sequence conservation on the protein surface around the Q entry point. There is also no proposal on how protons released from internal Q10 would lead to proton translocation. The authors also reiterate the assignment of the open and closed conformations to the deactive and active states, respectively, without providing any new data for this claim and using the same reasoning which we dismissed in a previous publication [32]. One of their main arguments is that the closed/open ratio does not change depending on redox conditions. As noted also in this review, the mammalian enzyme as purified exists with a certain energy profile resulting in a certain (variable) distribution of closed/open states in apo conditions. During turnover the enzyme is driven by redox reactions to transition between these states, resulting in proton pumping, but the overall energy profile does not have to change and so the closed/open distribution does not change. An analogous situation is seen with ATP synthase, where the distribution of rotary states does not change much between the apo and turnover conditions [68]. The main novel piece of data in [67] is the mode of Q10 binding, while the other structural findings are similar to those previously reported [32]. Therefore, there is no experimental basis for the mechanistic proposals in [67].

## Figures and Tables

**Table 1:** Core subunits of complex I.

Module	*Escherichia coli*	*Thermus thermophilus*	*Yarrowia lipolytica*	*Bos taurus* (Bovine)	*Homo sapiens*	
Peripheral arm Dehydrogenase (N)	**NuoF**	**Nqo1**	**NUBM**	**51 kDa**	**NDUFV1**	Cofactors^a^ FMN N3 (4Fe [51])
	**NuoE**	**Nqo2**	**NUHM**	**24 kDa**	**NDUFV2**	N1a (2Fe [24])
	**NuoG**	**Nqo3**	**NUAM**	**75 kDa**	**NDUFS1**	N1b (2Fe[75]) N4 (4Fe[75]C) N5 (4Fe[75]H) (N7)^b^
Connecting (Q)	**NuoD (NuoCD)^c^**	**Nqo4**	**NUCM**	**49 kDa**	**NDUFS2**	No cofactor
	**NuoC^c^**	**Nqo5**	**NUGM**	**30 kDa**	**NDUFS3**	No cofactor
	**Nuol**	**Nqo9**	**NUlM**	**TYKY**	**NDUFS8**	N6a (4Fe[TY]1) N6b (4Fe[TY]2)
	**NuoB**	**Nqo6**	**NUKM**	**PSST**	**NDUFS7**	N2 (4Fe[PS])
Membrane arm – Pumping (P)						TMH^d^
	**NuoH**	**Nqo8**	**NU1M**	**ND1**	**ND1**	8 – 9
	**NuoA**	**Nqo7**	**NU3M**	**ND3**	**ND3**	3
	**NuoJ**	**Nqo10**	**NU6M**	**ND6**	**ND6**	5
	**NuoK**	**Nqo11**	**NULM**	**ND4L**	**ND4L**	3
	**NuoN**	**Nqo14**	**NU2M**	**ND2**	**ND2**	11–14
	**NuoM**	**Nqo13**	**NU4M**	**ND4**	**ND4**	14
	**NuoL**	**Nqo12**	**NU5M**	**ND5**	**ND5**	16–17

a The traditional nomenclature for Fe-S clusters (Nx, derived from initially described electron paramagnetic resonance (EPR) signatures [65], as well as the nomenclature proposed [66] on the basis of re-assignment of EPR signals to structurally observed clusters, is shown. In the new nomenclature, clusters are named according to their nuclearity (2Fe or 4Fe), their subunit location (using bovine nomenclature) and when necessary, as ligated by four Cys (C) or three Cys and one His (H).

b Cluster N7 is present only in some bacteria (for example, *E*. *coli* and *T*. *thermophilus*).

c Subunits NuoC and NuoD are fused in *E*. *coli* and some other bacteria.

d Number of transmembrane helices.
